# Outcomes of primary orbital implantation in evisceration/enucleation in patients with endophthalmitis in a tertiary hospital

**DOI:** 10.1186/s12886-026-04871-0

**Published:** 2026-05-07

**Authors:** Saleh Alrashed, Maram Alnefaim, Rawan N. Althaqib, Hamad M. Alsulaiman

**Affiliations:** Oculoplastic and Orbit Division, King Khaled Eye Specialist Hospital, Riyadh, Kingdom of Saudi Arabia

**Keywords:** Endophthalmitis, Orbit, Primary orbital implantation, Evisceration, Enucleation, Anophthalmic socket, Postoperative complications, Implant exposure, Implant extrusion

## Abstract

**Purpose:**

To evaluate the safety and outcomes of primary orbital implantation during evisceration/enucleation for endophthalmitis, specifically analyzing the risk of implant exposure/extrusion.

**Methods:**

Retrospective review of 98 endophthalmitis patients (90 eviscerations, 8 enucleations) who received a primary orbital implant (mostly PMMA) over 10 years. Follow-up averaged 27.5 months.

**Results:**

Postoperative implant exposure/extrusion occurred in 10 patients (10.2%). This rate is comparable to established literature. The mean time to exposure was 140 days. Neither preoperative orbital cellulitis nor culture positivity (e.g., *Pseudomonas*) was statistically associated with a higher risk of exposure. Re-operations for exposure did not show evidence of active, ongoing infection.

**Conclusion:**

Primary orbital implantation in endophthalmitis patients is safe, carrying an exposure/extrusion risk (10.2%) comparable to reported rates. The procedure should be favored over a two-staged approach unless significant inflammation is noted intraoperatively.

**Précis:**

The purpose of this paper is to study outcomes of primary orbital implantation in Endophthalmitis patient, and to review the exposure rate, and to also analyze possible risk factors for the exposure/extrusion.

## Introduction

Endophthalmitis is a severe ophthalmic emergency that requires prompt and aggressive management. Current treatment modalities typically include intravitreal antibiotic injections, pars plana vitrectomy, or a combination of both. However, in cases refractory to these interventions, concern for uncontrolled intraocular infection and potential spread to the surrounding orbit or even intracranially may necessitate more definitive surgical management. In such circumstances, evisceration or enucleation becomes the optimal therapeutic approach. Historically, evisceration has been favored over enucleation when there is concern for potential subarachnoid dissemination of infection. Conversely, enucleation is considered more appropriate when the infection has significantly extended to involve the surrounding orbital tissues, rendering globe preservation or evisceration unsuitable [1]. The surgical management of endophthalmitis has traditionally advocated a two-staged approach to orbital implantation. This strategy involves performing evisceration or enucleation as the primary procedure and delaying orbital implant placement to a subsequent stage [2, 3]. This strategy was primarily intended to mitigate the risks of implant exposure and extrusion. A national survey from the United Kingdom revealed that only 43% of respondents would place an orbital implant in eyes undergoing enucleation or evisceration for endophthalmitis, and only 50% of these implants were placed primarily implants [4]. Similarly, a 17-question web-based survey distributed to surgeons from the American Society of Ophthalmic Plastic and Reconstructive Surgery (ASOPRS) indicated that 65% and 50% supported primary orbital implant placement at the time of evisceration and enucleation, respectively. However, the inherent drawback of a two-staged procedure is the prolongation of the patient’s recovery and rehabilitation period. Recent literature increasingly supports the acceptability of primary orbital implantation in patients with endophthalmitis, demonstrating a low incidence of implant exposure and extrusion in single-staged surgery [5, 6, 15, 7–14]. The aim of this paper is to study the outcomes of primary orbital implantation in patients undergoing evisceration or enucleation for endophthalmitis, and to analyze the risk factors contributing to implant exposure and extrusion.

## Method

This retrospective study was conducted at King Khalid Eye Specialist Hospital (KKESH), Riyadh, Saudi Arabia. Patient data were collected retrospectively over a 10-year period, from March 2014 to December 2023.

Cases were identified through surgical coding for evisceration and enucleation procedures performed at our institution. A total of 883 cases were initially identified, encompassing diverse surgical indications such as blind painful eye, blind painless eye, unsalvageable trauma, ocular malignancy, and endophthalmitis.

Of these, 98 cases of endophthalmitis were included for analysis. Exclusion criteria comprised non-endophthalmitis cases, cases with incomplete medical records, and endophthalmitis cases without primary orbital implant placement. Orbital implant exposure was defined as visualization of the implant through conjunctival dehiscence while remaining within the socket, whereas orbital implant extrusion was defined as complete displacement of the implant out of the orbital socket.

Documented variables included patient age, laterality of involvement, gender, relevant medical history, etiology of endophthalmitis, microbial culture results, orbital implant type and size, findings from preoperative imaging, date of presentation, date of surgery, date of last documented follow-up, and the occurrence and date of implant exposure or extrusion.

The diagnosis of endophthalmitis was primarily based on clinical presentation and imaging findings, further supported by microbial culture results. The decision to perform evisceration or enucleation was made by the primary treating ophthalmology team following medical and surgical management, including topical and intravitreal antibiotic administration and attempted pars plana vitrectomy.

This study received ethical approval from the Research Council and the Human Investigation Committee of King Khalid Eye Specialist Hospital, Riyadh, Saudi Arabia, and was conducted in accordance with the Declaration of Helsinki. The requirement for informed consent was waived for all adult patients and for the parents/legal guardians of pediatric patients after thorough explanation of the study procedures.

### Statistical Analysis

Data were collected, stored and management in a spreadsheet using Microsoft Excel 2010^®^ software. Data were coded and analyzed using SPSS^®^ version 21.0 (*IBM* Inc.^,^ Chicago, Illinois, USA). Descriptive analysis was done where categorical variables were presented as numbers and percentages, n (%) and continuous variables reported as mean ±standard deviation (SD) and range; this was after the normality tests (Shapiro-Wilk test and Q-Q plots) showed normally distributed data. Consequently, independent t-tests were used to test the differences between the groups’ means. For comparing the proportions Chi-squared test was done and chi squared or Fisher test p values reported appropriately. Any output with a *p* below 0.05 was interpreted as an indicator of statistical significance.

Surgical technique:

Evisceration procedures were predominantly performed under general anesthesia; local anesthesia was utilized only when the patient’s medical condition precluded general anesthesia. The eye and the periorbital area were prepared and draped in a sterile fashion. A 360° peritomy was performed with Westcott scissors. An incision was made at the superior limbus with a Bard-Parker number 11 blade, and the cornea was removed with Westcott scissors. A dialysis spatula was used to dissect the uveal tissues from the sclera. The inside of the scleral shell was scraped with a flat evisceration spoon, and bleeding vessels were controlled with bipolar cautery. Absolute alcohol was used to denature any remaining uveal tissues. Povidone and gentamicin were used to wash the sclera shell and were washed with balanced salt solution. Two anterior relaxing sclerotomies sclerotomies were done between the extraocular muscles using Westcott. When posterior sclerotomies was done, 4 posterior relaxing sclerotomies were done posterior and the optic nerve was disinserted. The implant size was estimated with a sizer. After the appropriate-sized implant was inserted, the scleral shell was closed with 5 − 0 Vicryl sutures. Tenon closure was achieved with several interrupted 5 − 0 Vicryl sutures. The conjunctiva was closed with 6 − 0 Vicryal sutures. A conformer of the appropriate size was inserted, and maxtriol ointment was instilled. Local anesthesia of 50–50 mixture of 2% lidocaine and 0.75% bupivacaine with epinephrine 1:100,000. Elastic bandage was used to keep a fluffy pressure dressing over the socket and was kept for 48–72 h. All patient were kept on topical maxtriol ointment and oral painkiller, none was given systemic steroids.

The enucleation we preformed the same initial steps described above. All four recti muscles were hooked tagged with double armed 5 − 0 vicryal suture before disinserting them, and the obliques muscles were cut. Optic nerve was cut with enucleation scissors and packing with epinephrine for 5 min was done. Appropriate size implant was placed, all implants were wrapped it in a scleral doner and secured it with 5 − 0 Vicryal. The implant was then introduced, and all four recti were secured with the same 5 − 0 Vicryal sutures. Final closure steps mirrored those of the evisceration procedure.

## Results

A retrospective review was conducted on 98 patients (98 eyes) who received a primary orbital implant. Of these, 8 patients underwent enucleation, while 90 underwent evisceration. The cohort comprised 56 males (57.1%) and 42 females (42.9%). Patient ages ranged from 2 months to 90 years. The average follow-up duration was 27.5 ± 28.5 months, and the average time from presentation to surgery was 27.1 ± 83.4 days (Table 1). Postoperative exposure or extrusion occurred in 10 patients (10.2%) (Table 2 ).

**Table 1:** Demographics and other characteristics

Characteristic	All (*n* = 98)	No extrusion (*n* = 88, 89.8%)	Extrusion/Exposure (*n* = 10, 10.2%)	*P* value
Age in years; mean ± SD [Range]	60.6 ± 19.7 [2 months – 90]	60.7 ± 19.8 [2 months – 90]	59.6 ± 20.3 [15–87]	0.868^£^
Gender				
Male, n (%)	56 (57.1)	51 (58.0)	5 (50.0)	0.630^α^
Female, n (%)	42 (42.9)	37 (42.0)	5 (50.0)	
Laterality				
OD, n (%)	44 (44.9)	39 (44.3)	5 (50.0)	0.732 ^α^
OS, n (%)	54 (55.1)	49 (55.7)	5 (50.0)	
Diabetes mellitus, n (%)	49 (49.0)	41 (46.6)	7 (70.0)	0.195^β^
Hypertension, n (%)	41 (41.8)	36 (40.9)	5 (50.0)	0.581 ^α^
Duration of follow-up, months mean ± SD [Range]	27.5 ± 28.5 [same day-114.6 months]	27.9 ± 29.1 [same day-114.6 months]	23.8 ± 22.4 [3.4–79.2]	0.665^£^
Duration between presentation and surgery, days, mean ± SD [Range]	27.1 ± 83.4 [same day-743 days]	21.4 ±42.0 [same day-225 days]	77.4 ± 233.9 [1-743]	0.469^£^

^£^Independent t-test; ^α^ Chi-squared test; ^β^Fisher’s exact test

**Table 2:** Comparing pseudomonas in the exposure and non-exposure groups

Group	*n* (%)	*P* value
No extrusion (*n* = 69)	8 (11.6)	0.140
Extrusion/Exposure (*n* = 10)	3 (30.0)	

The majority of orbital implants inserted were polymethyl methacrylate (PMMA). One patient received a hydroxyapatite implant, and another a silicone implant. The most frequently utilized orbital implant size was 18 mm (range: 8–20 mm, comprising 97.7% of implants of PMMA).

**Fig. 1:**
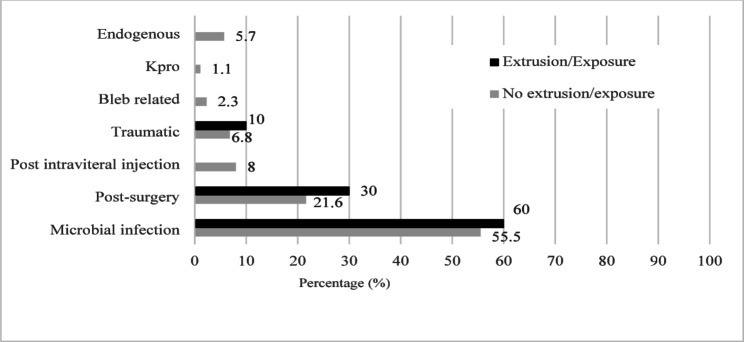
Cause of endophthalmitis

Regarding endophthalmitis, Microbial Keratitis was the most common etiology which was 55.5% (Fig. 1). Preoperative Computed Tomography (CT) scans that was done for 29 patients revealed orbital cellulitis in 19 patients, preseptal cellulitis in 8 patients, and no cellulitis in 7 patients. In the subset of patients who experienced postoperative exposure/extrusion, 2 had a 16 mm implant, 6 had an 18 mm implant, and 2 had a 20 mm implant. The causes of endophthalmitis within this exposure/extrusion group were attributed to: microbial infection (*n* = 6), surgical procedure (*n* = 3), and trauma (*n* = 1). Regarding the etiology of endophthalmitis, 93 cases were attributed to exogenous causes, while 5 cases were of endogenous origin. Notably, no endogenous cases were observed within the exposure/extrusion cohort (Tables 3 and 4). The mean duration from surgery to the onset of exposure was 140.3 ± 166.3 days (range: 5-479 days) (Table 5). Of the 98 patients included in this study, microbial cultures were performed in 41 (41.8%). Among these, 41 cultures (41.8%) yielded no growth. Pseudomonas aeruginosa was the most frequently isolated organism, identified in 8 cases (8.2% of the total cohort, or 19.5% of cultured cases), followed by Streptococcus pneumoniae in 4 cases (4.1% of the total cohort, or 9.8% of cultured cases).

**Table 3:** Distribution of the risk factors and extrusion rate

	No extrusion (*n* = 88)	Extrusion/Exposure (*n* = 10)	Extrusion/Exposure rate	*P* value
Implant size in mm, mean ± SD [Range]	17.5 ± 1.9	18.0 ± 1.3		0.371^£^
8 mm, n (%)	1 (1.1)	0 (0.0)	0.0	0.998^β^
12 mm, n (%)	1 (1.1)	0 (0.0)	0.0	0.998 ^β^
14 mm, n (%)	4 (4.5)	0 (0.0)	0.0	0.998 ^β^
16 mm, n (%)	20 (22.7)	2 (20.0)	9.1	0.998 ^β^
18 mm, n (%)	50 (56.8)	6 (60.0)	10.7	0.998 ^β^
20 mm, n (%)	12 (13.6)	2 (20.0)	14.3	0.632 ^β^
Cause of Endophthalmitis, n (%)				
Microbial infection	48 (55.5)	6 (60.0)	11.1	0.998 ^β^
Post-surgery	19 (21.6)	3 (30.0)	13.6	0.689 ^β^
Post intraviteral injection	7 (8.0)	0 (0)	0.0	0.998 ^β^
Traumatic	6 (6.8)	1 (10.0)	14.3	0.541 ^β^
Bleb related	2 (2.3)	0 (0)	0.0	0.998 ^β^
Kpro	1 (1.1)	0 (0)	0.0	0.998 ^β^
Non-endogenous	83 (94.3)	10 (100)	10.8	0.998 ^β^
Endogenous	5 (5.7)	0 (0.0)	0.0	
Preoperative CT finding, (n, %) (*n* = 34)				
Orbital cellulitis	16 (55.2)	3 (60.0)	15.8	0.402 ^β^
Preseptal cellulitis	7 (24.1)	1 (20.0)	12.5	0.592 ^β^
No cellulitis	6 (20.7)	1 (20.0)	14.3	0.541 ^β^
Implant type, (n, %)				
PMMA	86 (97.7)	10 (100)	10.4	0.998 ^β^
Hydroxyapatite	1 (1.1)	0 (0.0)	0.0	0.998 ^β^
Silicone	1 (1.1)	0 (0.0)	0.0	0.998 ^β^
Scleral Closure, (n, %)				
Single	83 (94.3)	9 (90.0)	9.8	0.485 ^β^
Double	5 (5.7)	1 (10.0)	16.7	
Tarasorraphy, (n, %)				
Yes	6 (6.8)	0 (0.0)	0.0	0.998 ^β^
No	82 (93.2)	10 (100)	10.9	

^£^Independent t-test; ^β^Fisher’s exact test; CT: computed tomography

**Table 4:** Patient characteristics on causes, implant type, size, surgery type, culture results and complications

Patient No.	Age (yrs)	Eye	Cause of Endophthalmitis	Implant size (mm)/Type	Type of Surgery	Cultures results	Duration from surgery to exposure (days)
1	62	Left	Microbial infection	16/PMMA	Evisceration	No growth	
2	73	Right	Microbial infection	12/PMMA	Evisceration	No growth	
3	79	Left	Post intraviteral injection	18/PMMA	Evisceration	Not done	
4	19	Right	Traumatic	18/PMMA	Evisceration	No growth	
5	72	Left	post-surgery	16/PMMA	Evisceration	Not done	
6	72	Right	Microbial infection	18/PMMA	Evisceration	Not done	
7	58	Left	Microbial infection	14/PMMA	Evisceration	Stains are negative/ culture not done	
8	90	Left	Microbial infection	14/PMMA	Evisceration	No growth	
9	67	Left	Microbial infection	18/PMMA	Evisceration	Streptococcus pneumonia	
10	61	Right	post-surgery	16/PMMA	Evisceration	No growth	8
11	56	Left	Microbial infection	20/PMMA	Evisceration	No growth	
12	60	Right	Microbial infection	18/PMMA	Evisceration	Not done	
13	41	Left	Microbial infection	18/PMMA	Evisceration	Pseudomonas aeruginosa	
14	77	Left	Microbial infection	18/PMMA	Evisceration	Not done	
15	70	Left	post-surgery	18/PMMA	Evisceration	Enterococcus faecialis	
16	78	Right	Post intraviteral injection	16/PMMA	Evisceration	Not done	
17	50	Left	post-surgery	16/PMMA	Evisceration	Pseudomonas aeruginosa	
18	59	Right	Microbial infection	18/PMMA	Evisceration	Not done	
19	54	Left	Microbial infection	18/PMMA	Evisceration	Not done	
20	50	Left	Microbial infection	18/PMMA	Evisceration	Not done	
21	76	Left	Post intraviteral injection	16/PMMA	Evisceration	MRSA	
22	55	Left	Microbial infection	18/PMMA	Evisceration	Pseudomonas	
23	78	Right	post-surgery	16/PMMA	Evisceration	Pseudomonas	
24	78	Right	Microbial infection	18/PMMA	Evisceration	Not done	
25	39	Left	Microbial infection	18/PMMA	Evisceration	Not done	
26	72	Left	Microbial infection	18/PMMA	Evisceration	Pseudomonas	
27	65	Right	post-surgery	16/PMMA	Evisceration	MRSA	
28	55	Left	Microbial infection	18/PMMA	Evisceration	No growth	
29	4 months	Right	Endogenous	8/PMMA	Evisceration	Escherichia coli	
30	58	Right	post-surgery	18/PMMA	Evisceration	No done	
31	67	Right	post-surgery	20/PMMA	Evisceration	No growth	
32	87	Right	Microbial infection	16/PMMA	Evisceration	MRSA	375
33	55	Left	Traumatic	18/PMMA	Evisceration	Not done	
34	60	Left	Microbial infection	20/PMMA	Evisceration	No growth	
35	73	Right	Kpro	18/PMMA	Evisceration	No growth	
36	28	Right	Microbial infection	18/PMMA	Evisceration	No growth	
37	58	Left	post-surgery	18/PMMA	Evisceration	No growth	
38	65	Left	Microbial infection	18/PMMA	Evisceration	No growth	
39	55	Left	Bleb related	20/PMMA	Evisceration	haemophilus influenzae	
40	61	Left	post-surgery	18/PMMA	Evisceration	Streptococcus mitis	
41	61	Right	post-surgery	18/PMMA	Evisceration	No growth	
42	51	Right	Microbial infection	18/PMMA	Evisceration	No growth	
43	68	Right	Post intraviteral injection	18/PMMA	Evisceration	No growth	
44	65	Right	Microbial infection	16/PMMA	Evisceration	Streptococcus pyrogens	
45	73	Left	Microbial infection	16/PMMA	Evisceration	Not done	
46	88	Right	Microbial infection	18/PMMA	Evisceration	No growth	
47	47	Left	post-surgery	18/PMMA	Evisceration	Streptococcus salivarius	
48	71	Right	Traumatic	20/PMMA	Evisceration	No growth	
49	48	Left	Microbial infection	18/PMMA	Evisceration	Moraxella / micrococcus luteus	
50	70	Left	Microbial infection	18/PMMA	Evisceration	Not done	
51	71	Right	Microbial infection	18/PMMA	Evisceration	Not done	
52	66	Left	post-surgery	18/PMMA	Evisceration	Not done	
53	57	Right	Post intraviteral injection	18/PMMA	Evisceration	Not done	
54	71	Right	post-surgery	18/PMMA	Evisceration	Streptococcus pneumonia	5
55	71	Right	post-surgery	18/PMMA	Evisceration	No growth	
56	39	Right	Traumatic	20/PMMA	Evisceration	None	
57	67	Left	Microbial infection	18/PMMA	Evisceration	None	221
58	6	Right	Bleb related	20/PMMA	Evisceration	Streptococcus pneumonia	
59	68	Left	Microbial infection	16/PMMA	Evisceration	Pseudomonas aeruginosa	
60	65	Left	Microbial infection	18/PMMA	Evisceration	None	
61	61	Left	post-surgery	20/PMMA	Evisceration	Streptococcus mitis/oralis	
62	57	Left	Microbial infection	18/PMMA	Evisceration	None	
63	77	Left	Microbial infection	18/PMMA	Evisceration	Pseudomonas aeruginosa	58
64	73	Right	Post intraviteral injection	18/PMMA	Evisceration	None	
65	83	Left	Microbial infection	20/PMMA	Evisceration	Streptococcus mitis/oralis	
66	83	Left	Microbial infection	18/PMMA	Evisceration	Streptococcus mitis/oralis	
67	40	Left	Microbial infection	20/PMMA	Evisceration	Pseudomonas aeruginosa	
68	15	Left	Traumatic	20/PMMA	Evisceration	Bacillus species	135
69	41	Left	Microbial infection	18/PMMA	Evisceration	None	40
70	60	Right	Endogenous	18/PMMA	Enucleation	None	
71	12	Left	Microbial infection	16/hydroxyapatite	Enucleation	staphylococcus aureus	
72	5	Right	Traumatic	18/PMMA	Enucleation	None	
73	87	Left	Microbial infection	18/PMMA	Enucleation	Pseudomonas aeruginosa	
74	67	Left	post-surgery	18/PMMA	Enucleation	No growth	
75	17	Right	Microbial infection	18/PMMA	Enucleation	None	
76	83	Right	post-surgery	18/PMMA	Enucleation	staphylococcus aureus	
77	87	Right	post-surgery	18/PMMA	Evisceration	None	
78	79	Right	post-surgery	18/PMMA	Evisceration	None	
79	78	Right	Microbial infection	14/PMMA	Evisceration	rothia mucilaginosa	
80	61	Right	Post intraviteral injection	16/PMMA	Evisceration	None	
81	71	Left	Microbial infection	16/PMMA	Evisceration	Streptococcus pneumonia	
82	78	Right	Microbial infection	18/PMMA	Evisceration	none	
83	63	Right	post-surgery	18/PMMA	Evisceration	Pseudomonas aeruginosa	31
84	86	Left	Microbial infection	16/PMMA	Evisceration	streptococcus pluranimalium	
85	66	Left	Microbial infection	16/PMMA	Evisceration	None	
86	2 months	Left	Endogenous	16/PMMA	Evisceration	salmonella	
87	64	Left	Microbial infection	20/PMMA	Evisceration	None	479
88	51	Left	post-surgery	16/PMMA	Evisceration	None	
89	52	Right	Microbial infection	18/PMMA	Evisceration	None	
90	72	Left	Microbial infection	16/PMMA	Evisceration	None	
91	66	Right	Microbial infection	18/PMMA	Evisceration	None	
92	50	Right	Microbial infection	18/PMMA	Evisceration	Pseudomonas aeruginosa	51
93	76	Right	Traumatic	16/PMMA	Evisceration	Escherichia coli	
94	54	Left	Endogenous	16/PMMA	Evisceration	klebsiella pneumoniae	
95	56	Left	Endogenous	14/PMMA	Evisceration	Staphylococcus aureus	
96	78	Left	Microbial infection	20/silicone	Enucleation	None	
97	58	Right	Microbial infection	20/PMMA	Evisceration	Not done	
98	74	Left	Microbial infection	18/PMMA	Evisceration	No growth	

**Table 5:** Duration from surgery to exposure in days (*n* = 10)

	Mean ± SD [Range]
Duration from surgery to exposure (days)	140.3 ± 166.3 [5-479]

Within the subgroup of patients experiencing postoperative exposure or extrusion, positive cultures revealed the following: Pseudomonas aeruginosa (*n* = 3), Streptococcus pneumoniae (*n* = 1), Bacillus species (*n* = 1), and Methicillin-resistant Staphylococcus aureus (MRSA) (*n* = 1). The remaining cultures from this subgroup showed no growth (Table 6).

**Table 6:** Culture results

Organism	*n* (%)
No growth	41 (41.8)
Pseudomonas aeruginosa	8 (8.2)
Streptococcus pneumonia	4 (4.1)
MRSA	3 (3.1)
Pseudomonas	3 (3.1)
Staphylococcus aureus	3 (3.1)
Streptococcus mitis/oralis	3 (3.1)
Escherichia coli	2 (2)
Bacillus species	1 (1)
Enterococcus faecialis	1 (1)
Haemophilus influenzae	1 (1)
Klebsiella pneumoniae	1 (1)
Moraxella / micrococcus luteus	1 (1)
Rothia mucilaginosa	1 (1)
Salmonella	1 (1)
Stains are negative/ culture not done	1 (1)
Streptococcus mitis	1 (1)
Streptococcus pluranimalium	1 (1)
Streptococcus pyrogens	1 (1)
Streptococcus salivarius	1 (1)
Not done	19 (19.4)

## Discussion

Endophthalmitis is a severe ophthalmic emergency that frequently necessitates enucleation or evisceration to prevent widespread ocular and potential systemic complications. The practice of delaying orbital implantation in these patients to reduce the risk of exposure or extrusion remains controversial.

Historical perspectives and some studies have advocated for a two-staged approach, wherein orbital implantation is deferred to a subsequent procedure to minimize postoperative complications. Conversely, a growing body of recent literature supports the feasibility and safety of primary orbital implantation at the time of initial evisceration or enucleation for endophthalmitis. Dresener et al. evaluated 11 patients with endophthalmitis who underwent primary orbital implantation following evisceration, although no specific follow-up duration was reported. Only 1 patient (9.09%) developed implant exposure postoperatively [15]. In 2005, Liu published a study on primary orbital implantation in patients with endophthalmitis that included both prospective and retrospective analyses. In the prospective arm, 25 patients with endophthalmitis were followed for an average of 42 months. Remarkably, none of these patients experienced orbital implant exposure or extrusion. The retrospective review included 37 primary orbital implantation procedures performed by his colleagues. Although the follow-up duration was not explicitly stated, it was reported to average approximately 36 months. Among these 37 procedures, 10 patients (27%) experienced postoperative extrusion. [6] n 2007, Ozgur and colleagues conducted a review of 25 patients who underwent evisceration with primary orbital implantation for endophthalmitis. Patients in this series were followed for an average duration of 24 months [16]. Their findings indicated that 3 patients (12%) experienced postoperative implant exposure [16]. In the same year, Tawfik and Budin conducted a study on 66 endophthalmitis patients who underwent primary orbital implantation [8]. With an average follow-up period of 11 months, their study reported that 3 patients (4.5%) experienced postoperative implant exposure [8].

In Liu’s paper, delayed orbital implantation was also evaluated in 15 patients with endophthalmitis, with a follow-up duration of 24 months [6]. None of the patients experienced implant exposure or extrusion during the follow-up period.

In 2005, Gaillard et al. reported four patients with endophthalmitis managed with a staged approach, consisting of initial evisceration followed 4–6 weeks later by enucleation and placement of an alumina implant wrapped in autologous sclera. During a follow-up period of 10–32 months, no exposures or extrusions were observed, supporting the safety of delayed secondary implantation in selected cases [17]. In 2015 Francesco and colleagues studied a total of 86 patients with delayed orbital implantation from various causes retrospectively [18]. Their patient were divided into two groups, 41 were of enucleation and 45 were of evisceration and were followed up at least 2 years, they noted only two patients had exposure and that was only in the enucleation group While delayed implantation has very low reported exposure, the evidence is limited to small case series. In our study, which was comparable to the previously published studies, we reviewed 98 with endophthalmitis and primary orbital implantation patients with an average duration of a follow-up of 27 months. Only 10 patients (10.2%) had exposure/extrusion during the follow-up period, which is comparable to the previously published papers. This falls within the range of most previous papers (4.5%-24%).

The questions arise if the exposure rate in endophthalmitis patients is comparable to other post evisceration cases. In 2023 Kenneth Ka Hei Lai et al. reviewed 79 patients who had undergone evisceration, they compared endophthalmitis patient to non-endophthalmitis patients with an average duration of follow up of 74 ± 46 months. Of their 79 patients, 26 were of the endophthalmitis group. In the exposure/extrusion group, they reported a statistical difference of high risk of exposure/extrusion in the endophthalmitis group (54% vs. 17%, *P* < 0.05) [19]. Wongsaithong El al. looked at exposure/extrusion in a 11-year-study. The indication in their study were of, infection, trauma, tumor and other causes. In total, they were able to collect 466 patients. The orbital implant exposure/extrusion rate was 12.14% (38/313) of patients in the enucleation group and 17.65% (27/153) in the evisceration group^20^. In 2007 Chaudhry et al. reviewed 187 patients in our institute in a 3-year period, to evaluate the indications of evisceration and their resultant complications. The mean duration of follow up is 15 months with a reported exposure rate of 10 (5.7%) [21]. (Table 7 )

**Table 7:** Comparison to Authors with similar articles

Author / Year	Type of Study	Number of Patients	Follow-up Duration	Exposure / Extrusion Rate
Dresner et al. 2005	Case series (primary)	11 (endophthalmitis)	NR	1/11 (9.1%)
Liu, 2005 (prospective)	Prospective cohort (primary)	25	42 months (mean)	0%
Liu, 2005 (retrospective)	Retrospective cohort (primary)	37	~ 36 months (mean)	10/37 (27%)
Ozgur et al., 2007	Retrospective review (primary)	25	24 months (mean)	3/25 (12%)
Tawfik & Budin, 2007	Retrospective review (primary)	66	11 months (mean)	3/66 (4.5%)
Author’s sttudy	Retrospective cohort (primary)	98	27 months (mean)	10/98 (10.2%)
Liu, 2005 (delayed)	Prospective cohort (delayed)	15	24 months (mean)	0%
Gaillard et al., 2005	Case series (delayed)	4	10–32 months	0%
Francesco et al., 2015	Retrospective (delayed, mixed causes)	86	≥ 24 months	2 cases (only enucleation group)

There have been reports of risk factors that increase the rates of exposure postopertive in endophthalmitis patients. Many authorities believe that eyes infected with a virulent protease-secreting organism, such as Pseudomonas, are at higher risk of implant extrusion [4, 15, 22]. However, we found no difference in our study. In 2010, Kim Et published a paper to better understand the risk factors of exposure in endophthalmitis patients [11]. They used Multiple logistic regression analysis for their results, and what they found was orbital cellulitis as the only risk factor. In our study, we had 16 patients of orbital cellulitis in the non exposure/extrusion group and only 3 in the exposure/extrusion group. No statistical difference was found between them. We believe that complete control of the infection in these patients was done preopertive, intraopertive, and postopertive with either receiving oral or intravenouns antibiotics. In reviewing the cases of patients who developed postoperative exposure or extrusion, we conducted a detailed analysis of their medical records to identify potential contributing factors. Patient ages ranged from 15 to 87 years, with a gender distribution of four males and five females. All patients received PMMA implants, ranging in size from 16 to 20 mm. Management strategies varied and included implant removal without replacement—often at the patient’s request—dermis fat grafting, or secondary implantation with a smaller implant. Notably, during reoperation, none of these patients exhibited signs of active infection related to the initial endophthalmitis. Therefore, we believe that the cause of implant exposure in these cases was most likely unrelated to ongoing or unresolved infection.

The limitation of this study is that it is a retrospective study, with no comparison of the non-endophthalmitis patients. The surgeries in this study were performed by multiple surgeons and not by one surgeon. There was no comparison to delayed orbital implantation, or secondary procedure like dermis fat grafting.

## Conclusion

Primary orbital implantation at the time of evisceration or enucleation for endophthalmitis appears to be a safe and effective option, with an implant exposure/extrusion rate comparable to previously published series. In this study, postoperative complications were uncommon, and no clear association was identified between exposure/extrusion and traditionally presumed risk factors such as positive microbial cultures or preoperative orbital cellulitis. These findings support the growing evidence that delaying implantation may not be necessary in appropriately selected patients and that a single-stage approach can facilitate earlier socket rehabilitation while avoiding the burden of a second procedure. However, when adequate wound closure cannot be achieved intraoperatively because of poor tissue integrity, excessive tension, or technically difficult suturing, postponing orbital implantation may be the more prudent option to reduce the risk of postoperative exposure or extrusion. Further prospective comparative studies are warranted to better define patient selection criteria and to directly compare outcomes with delayed orbital implantation.

## Data Availability

No datasets were generated or analysed during the current study.
